# Alpha-Hemoglobin-Stabilizing Protein: An Erythroid Molecular Chaperone

**DOI:** 10.1155/2011/373859

**Published:** 2011-03-24

**Authors:** Maria Emília Favero, Fernando Ferreira Costa

**Affiliations:** ^1^Department of Pathology, Clinical Analysis and Toxicology, University Hospital, State University of Londrina (UEL), Avenida Robert Koch, 60, Vila Operária, 86038-350 Londrina, PR, Brazil; ^2^Hematology and Hemotherapy Center, State University of Campinas (UNICAMP), Rua Carlos Chagas, 480, Cidade Universitária, Barão Geraldo, 13083-970, Campinas SP, Brazil

## Abstract

Alpha-hemoglobin-stabilizing protein (AHSP) is an erythroid-specific protein that acts as a molecular chaperone for the free *α* chains of hemoglobin. Evidence strongly suggests that AHSP participates in hemoglobin synthesis and may act to neutralize the cytotoxic effects of excess free alpha-globin subunits that accumulate both in normal and beta-thalassemic erythroid precursor cells. As such, AHSP seems to be essential for normal erythropoiesis, and impaired upregulation of AHSP may lead to premature erythroid cell death, resulting in ineffective erythropoiesis. Reduced *AHSP* mRNA expression has been associated with clinical variability in some cases of *β*-thalassemia. It has been shown that *α*Hb variants may also impair AHSP-*α*Hb interactions, leading to pathological conditions that resemble *α*-thalassemia syndromes. The aim of this paper is to summarize current information concerning the structure and function of AHSP, focusing on its role in normal erythropoiesis and its relevance in health and disease.

## 1. Introduction

The term “molecular chaperone” was first used in 1978 to describe the *in vitro* properties of nucleoplasmin, which prevents the aggregation of folded histone proteins with DNA during the assembly of the nucleosome [1]. However, the concept of the molecular chaperone as a protein that can interact with other proteins and help them to reach their final, active conformation was established only in 1987 [2]. Molecular chaperones are currently defined as “proteins whose role is to mediate the folding of certain other polypeptides and, in some instances, their assembly into oligomeric structures, but which are not components of these final structures” [3]. As such, molecular chaperones participate in a variety of physiological processes related to the correction of the structure of proteins during their synthesis and after their release from ribosomes, and more than 25 different protein families described as chaperones have been reported [4].

Alpha-hemoglobin-stabilizing protein (AHSP) is a small protein that binds specifically to the free alpha globin chain (*α*Hb) of hemoglobin (Hb). The main function of AHSP appears to be to regulate the stability, folding, and assembly of the *α*Hb subunit. The biochemical properties of the AHSP-*α*Hb complex led to the classification of AHSP as a novel type of chaperone. Like the small heat shock proteins (sHSPs), a family of molecular chaperones, AHSP presents low molecular weight and ATP-independent chaperone-like activity. However, unlike other molecular chaperones, it does not form oligomeric complexes and is highly tissue and substrate specific [5, 6]. AHSP plays an important role in the physiological process of erythropoiesis [5, 7] and may also be a modulator of pathological conditions [8–11]. Since chaperone proteins are essential for native protein conformation and defective chaperone proteins may be associated with diseases, the aim of this paper is to summarize the current research concerning the structure and function of AHSP, focusing on its role in normal erythropoiesis and its possible importance as a modifier of *β*-thalassemia.

## 2. Hemoglobin: Function and Biosynthesis

Hemoglobin is an essential protein for life, since it is responsible for binding oxygen in the lungs and delivering it to all tissues and organs in the body in a well-controlled manner. The major functional adult human Hb (HbA) is a heterotetramer composed of two alpha- (*α*Hb) and two beta- (*β*Hb) globin chains, each containing a prosthetic group-Heme [12].

Biosynthesis of hemoglobin occurs exclusively in cells of erythroid lineage and, therefore, is intimately related to erythropoiesis. Hemoglobin synthesis begins at the proerythroblast phase, continues at a high rate throughout the basophilic and the polychromatophilic erythroblast stages, and persists at a very low rate in the reticulocytes. This multistep process is complex and controlled by two distinct gene clusters: the *α*-like globin cluster located on chromosome 16 and the *β*-like globin cluster on chromosome 11; and is highly regulated to ensure that *α*Hb and *β*Hb are produced at a stoichiometric rate. During the first step of HbA assembly, *α*Hb and *β*Hb chains combine to form the *α*_1_ *β*_1 _interface, which involves hydrophobic and electrostatic interactions. In a second step, two dimers associate to form the *α*_1_ *β*_2_ interface, which involves more polar interactions [12]. Isolated *α*Hb is different from *β*Hb monomers in structural stability, where *β*Hb subunits are more stable than *α*Hb. After covalent association with heme, *β*Hb chains self-associate to form homotetramers (*β*_4_), called HbH. However, these tetramers are not functional due to their high affinity for oxygen. During auto-oxidation, *α*Hb monomers tend to produce reactive oxygen species (ROS) via chemical reactions catalyzed by heme-iron [13]. Free *α*Hb monomers are highly unstable and do not self-associate to form tetramers but tend to aggregate and precipitate, resulting in damage to the membrane and triggering the apoptosis of erythroid precursors (ineffective erythropoiesis) and a shortened lifespan of circulating red blood cells [14, 15].

Before the discovery of AHSP protein, it was postulated that the nascent polypeptide chains readily associate to form dimers (*αβ*), and soon after, to form tetramers (*α*_2_ *β*_2_) by a spontaneous process requiring no other molecules [16, 17]. This concept changed after Kihm and colleagues [6] showed that the small protein AHSP, encoded by a gene first described as *EDRF* [18], could form a complex with free *α*Hb, preventing its precipitation and neutralizing its deleterious effects. Currently, AHSP is known to play an important role in hemoglobin assembly and could have medical implications, since imbalances in globin synthesis are associated with serious human diseases [14]. In thalassemia disorders, mutations in the *α*-globin (*HBA*) or *β*-globin (*HBB*) genes lead to an excess of free *β*Hb or *α*Hb subunits, respectively. Due to the great variability in *β*-thalassemia phenotype and the role of AHSP in preventing the cytotoxic effects of free *α*Hb in excess, it has been suggested that AHSP is a potential modifier of this disease.

## 3. AHSP: An Erythroid Molecular Chaperone

AHSP is a protein expressed in hematopoietic tissues (bone marrow in humans and fetal liver, spleen, and bone marrow in mice) and exclusively by cells of the erythroid lineage [6, 18]. AHSP forms a stable but reversible complex with free *α*Hb to stabilize its structure and limit its chemical reactivity. This interaction prevents *α*Hb aggregation and precipitation in solution and in live cells and therefore maintains *α*Hb available for incorporation into HbA [6, 19]. The AHSP-*α*Hb interaction also inhibits ROS production and, therefore, oxidative damage [20, 21]. AHSP does not bind to the *β*Hb subunit or tetrameric HbA. Indeed, structural analysis of AHSP-*α*Hb complex revealed that AHSP and *β*Hb compete to bind to *α*Hb in the same region, called the *α*_1_ *β*_1_ interface [5]. *β*Hb affinity to *α*Hb is stronger and wins this competition, displacing AHSP to form the HbA molecule. Consistent with the role of a molecular chaperone, in addition to its ability to stabilize *α*Hb, AHSP is also able to promote refolding of denatured *α*Hb [22].

## 4. AHSP: Gene Structure and Expression

The *AHSP* gene is located on human chromosome 16 (GenBank accession number AC106 730.2). The genomic structure of *AHSP* is organized in three exons and two introns, comprising approximately 1.8 kb. The *AHSP* mRNA encodes a protein of 102 amino acids with a molecular mass of 12 kDa; the protein is highly conserved in humans, pigs, cows, and rats [6].

Identification and characterization of the regulatory elements that control *AHSP* gene expression have important implications for normal erythropoiesis and the pathogenesis of hemolytic disorders. Like many other genes that participate in hemoglobin synthesis, *AHSP* gene expression is controlled by GATA-1 [6], a transcription factor that is essential for the survival and maturation of lineage-committed erythroid precursors. There is evidence that Oct-1 [23] and EKLF [24–26], two transcription factors that have a critical role in erythropoiesis, also participate in the control of *AHSP* gene expression.

The *AHSP* gene promoter comprises the region from −170 to +269 and includes the 5′-flanking DNA and intron 1. There are 5 consensus GATA-1 binding sites in this minimal promoter region (−179/+290), three in the 5′-flanking region and two in intron 1. An Oct-1 consensus site also exists in the AT-rich region of *AHSP* intron 1 [23]. EKLF directly regulates the *AHSP* gene by binding to a site at −60 in the human *AHSP* promoter region that is situated between two conserved GATA-1 binding sites at −47 and −90. Keys and colleagues [25] proposed that GATA-1 and EKLF are coregulators of *AHSP* gene expression during erythropoiesis.

Molecular analysis of the *AHSP* gene has identified several single nucleotide polymorphisms (SNPs) and gene haplotypes [9, 10, 27]. An SNP within the intron 1 of *AHSP* (12391 G > A SNP) alters the binding site for Oct-1, and there is evidence that this SNP can reduce *AHSP* expression *in vivo* [10]. Human *AHSP* gene expression can be affected by the iron status via an iron sensor system, composed of iron responsive elements (IREs) and iron regulatory proteins (IRP). A stem-loop structure that resembles the IRE was identified in the 3′UTR of *AHSP* mRNA. It has been shown that these IRE-like structures can interact with IRPs and that the IRE-IRP interactions can be disrupted by iron, resulting in destabilization of *AHSP* mRNA. These data suggest that *AHSP* gene expression is downregulated in the excess of iron and upregulated during iron deficiency [28].

## 5. AHSP: Protein Structure and Mechanism of Action

The structure of AHSP was determined by nuclear magnetic resonance spectroscopy (NMR) and X-ray crystallography [20, 21, 29]. In its native state, AHSP is comprised of a bundle of three elongated antiparallel *α*-helices and is conformationally heterogeneous. AHSP exists in two forms, cis and trans, that are distinguished because of the arrangements of the loop region between helices 1 and 2 due to cis/trans isomerization of residue Pro30. However, once AHSP binds to *α*Hb, Pro30 adopts just one conformation [29].

AHSP forms a simple heterodimer with *α*Hb via the *α*_1_ portion of the *α*_1_ *β*_1_ interface in HbA [5], which is the same interface as that of the *β*Hb interaction. The intermolecular contacts between *α*Hb and AHSP are less extensive than those between *α*Hb and *β*Hb and this may explain why AHSP is replaced by *β*Hb [11]. AHSP binds apo-*α*Hb (without heme) and holo-*α*Hb (with heme iron), either in the oxidized (FeIII) or reduced (FeII) states [5, 6, 20, 21]. Data suggest that the interaction of wild-type AHSP (AHSP^WT^) with nascent *α*Hb promotes correct folding into a native state, which is independent upon heme, conferring protection from proteolytic digestion. Consistent with the role of a molecular chaperone, AHSP also promotes refolding of denatured apo-*α*Hb [22]. 

Reports indicate that binding of AHSP to oxy-*α*Hb (FeII) is associated with structural alterations in the heme pocket. These initial changes are reversible and result in a structure that exposes the oxygen-binding site [21, 30]. If the oxy-*α*Hb-AHSP interaction persists, the structure of the heme pocket is altered further, auto-oxidation occurs and the heme-bond iron is converted to a ferric form in a Fe(III) bis-histidyl complex. This hemichrome structure inhibits the reaction of Fe(III)-*α*Hb with oxidants such as hydrogen peroxide, conferring resistance to hemin loss and denaturation. AHSP, bound to both the oxidized (ferric) and reduced (ferrous) *α*Hb forms, can be replaced by oxy-*β*Hb to generate functional HbA [30]. These data suggest that, in the absence of *β*Hb, AHSP maintains the excess *α*Hb in a reversible inert state to limit the production of damaging ROS.

The kinetics of *α*Hb binding-dissociation to AHSP was investigated by Mollan et al. [31]. The authors used the fluorescence energy transfer technique to study the rate of association and dissociation of the AHSP-*α*Hb complex. The *α*Hb and *β*Hb chains do not fluoresce due to high quenching of tryptophan or tyrosine fluorescence by the heme group, an efficient energy acceptor. AHSP has no heme group and demonstrates an intrinsic fluorescence typical of an exposed tryptophan at position 44. As such, quenching of fluorescence of the AHSP by binding to *α*Hb was used to measure the rate of AHSP-*α*Hb complex formation. Starting the experiment with isolated *α*Hb and AHSP proteins, these authors found that the apparent association rate constant for AHSP to *α*Hb (*k*′ ≈ 10 *μ*M^−1^ s^−1^) is greater than the association rate constant for (deoxy)*α*Hb to (deoxy)*β*Hb (*k*′ ≈ 0.5 *μ*M^−1^ s^−1^) in *in vitro* experiments. The *β*Hb chains were added to follow the displacement of *α*Hb from AHSP, and the increase in fluorescence intensity was used to detect the extent of AHSP dissociation. The dissociation equilibrium constant (*K*_*d*_) of *∼*10^−2^ *μ*M for the AHSP-*α*Hb complex was compared with the *K*_*d*_ of *∼*10^−6^ *μ*M for the *α*Hb-*β*Hb dimer. Thus, these data show that AHSP binds faster to *α*Hb than *β*Hb (*∼*20 times) and also that AHSP has a lower affinity for *α*Hb when compared with the affinity of *β*Hb for *α*Hb (*∼*10.000 times). These kinetic properties of AHSP are in agreement with its chaperone function, that is, fast binding to free *α*Hb to prevent its degradation and rapid release of *α*Hb for Hb assembly [31].

## 6. AHSP in Normal Erythropoiesis

The main characteristic of erythropoiesis is the massive synthesis of hemoglobin. As stated earlier, *α*Hb and *β*Hb subunits must be produced in equilibrium to prevent their harmful effects. However, there is experimental evidence that *α*Hb is produced in slight excess during normal erythropoiesis. The identification of the *AHSP* gene by Miele et al. [18] and the recognition of its ability to specifically bind to free *α*Hb by Kihm et al. [6] highlighted a new homeostatic mechanism to handle the harmful excess of free *α*Hb during HbA production.

Using a murine model, Kihm et al. [6] reported the first evidence that AHSP plays an important role in *in vivo* erythropoiesis. Transgenic mice, in which the entire protein- coding region of *Ahsp* gene (*Ashp* ^−/−^) was ablated, exhibited hematological alterations consistent with ineffective erythropoiesis that were similar to those observed in *β*-thalassemia. Unexpected results were obtained in a murine model of compound mutants lacking both *Ahsp* genes (*Ashp* ^−/−^) and 1 of 4 *α-Hb* genes (*α*Hb^−*α*/*αα*^); these mice exhibit a more anemic phenotype than expected. These findings suggest that AHSP plays an important role in erythropoiesis even when the pool of free *α*Hb is not present [22], probably because AHSP stabilizes newly synthesized *α*Hb to augment its incorporation into HbA. 

The expression of the *AHSP* gene during human erythropoiesis was first reported by Dos Santos et al. [7]. Differentiation of erythroid progenitor cells from healthy volunteers was achieved by a two-phase liquid culture system, stimulated with erythropoietin. Cell surface marker expression was accompanied during cellular differentiation using flow cytometry and by morphological analyses. Quantification of *AHSP*, *HBA*, and *HBB* gene expression was determined by the real time polymerase chain reaction at four distinct stages of erythropoiesis: during the earlier phase in which proerythroblasts begin to be discernible (3-4 days of phase II); during the intermediate phase characterized by the appearance of basophilic erythroblasts (5–7 days) and an increasing proportion of polychromatic and orthochromatic erythroblasts (8–10 days), and during the late phase in which orthochromatic erythroblasts predominate (11–14 days). Consistent with erythropoiesis physiology, higher levels of *HBA* expression were observed during the intermediate phase, in which polychromatic and orthochromatic erythroblasts predominate, and it was followed by a decrease in more mature precursors, present during the late phase. Interestingly, *AHSP* expression during normal erythropoiesis showed a pattern similar to that of *HBA*, with the highest expression at the stage where hemoglobin biosynthesis is more intense. These data demonstrated that the ratio between *AHSP* and *HBA* expression is conserved in all stages of normal erythropoiesis and strongly suggest that AHSP plays an important role in human erythropoiesis [7]. 

To better understand the relationship between AHSP expression, HbA formation, and the consequences of a reduced AHSP synthesis in human red blood cells, an RNA-interference knockdown of AHSP expression was established both in hemin-induced K562- cells and human hematopoietic stem cells (CD34^+^). Decreased AHSP expression levels were observed in response to AHSP knockdown in both K-562 and EPO-induced CD34^+^ cells. The decrease in *AHSP* expression was associated with a remarkable *α*Hb precipitation in erythroid precursors, when compared with negative control cells. Significant increases in ROS production and cell death were also observed [19]. These findings were in agreement with the data obtained in models of transgenic mice [6, 8] and provided further evidence that AHSP plays an important role in human erythropoiesis. Data from *in vitro* and *in vivo* studies suggest that AHSP participates in HbA formation by both preventing *α*Hb precipitation and degradation or by enhancing proper folding and heme insertion [22, 32–34].

## 7. AHSP as a *β*-Thalassemia Modifier

Although *β*-thalassemia results from reduced *β*Hb synthesis, the pathophysiology of this disease is determined by the level of free *α*Hb chains that cause oxidative damage of red blood cells. The marked variability in clinical severity of *β*-thalassemia has been attributed to environmental and genetic factors, including hereditary persistence of fetal hemoglobin (HPFH) and coexistence of *α*-thalassemia [35]. However, these factors cannot explain the phenotypic variability in many cases of *β*-thalassemia.

Considering the biological and biochemical properties of AHSP, it is natural to speculate those AHSP mutations, leading to its reduced expression or altered function, could modify the clinical characteristics of *β*-thalassemia. Galanello et al. [36] provided the first evidence that AHSP could modulate *β*-thalassemia. They reported the association of reduced *AHSP* mRNA expression and a more severe phenotype among individuals with identical *β*-thalassemia genotypes in two unrelated Sardinian families. Observations from a murine model of *β*-thalassemia intermedia provided further evidence for this possibility. The phenotype of transgenic mice with *β*-thalassemia intermedia is exacerbated by concomitant AHSP loss [8]. *AHSP* mutations in *β*-thalassemia patients are uncommon and, to date, the association between *AHSP* mutation and *β*-thalassemia variability has not been well established; however, there exist some reports showing the association between *β*-thalassemia severity and *AHSP* genotype [10, 27, 37]. 

The investigation of AHSP sequence in healthy and *β*-thalassemia individuals from five different regions of the world identified a single nucleotide change in the third exon of the *AHSP* gene, resulting in a protein with an isoleucine at position 75, instead of an asparagine (AHSP^N75I^). This uncommon missense mutation does not affect the affinity of AHSP for *α*Hb and the oxidation of the ferric oxy (FeII)-*α*Hb to the ferrous (FeIII) form is induced at a similar rate, when compared to the AHSP^WT^. However, AHSP^N75I^ showed a reduced ability to inhibit ROS production by *α*Hb. Thus, there is evidence that the mutant protein is less effective against the oxidative effects of free *α*Hb. This mutation demonstrated clinical consequences in a patient, found to be compound heterozygous for AHSP^N75I^ and *β*-thalassemia (*β* ^0^ Codon 39 C > T), who presented with a severe anemia that required blood transfusion. However, when inherited without any thalassemia mutation, the heterozygous state does not show clinical relevance [10, 38]. 

Two other missense mutations, AHSP^D29V^ (12750 A > T) and AHSP^V56G^ (12831 T > G) were identified in a populational study to assess the effect of AHSP on the phenotype of *β*-thalassemia patients of Southern China, a region with a high prevalence of this genetic condition. However, neither of these mutations were associated with significant phenotypic variability among patients [39]. 

Lai et al. identified several sequence variants, including a Tn-homopolymer in the AHSP promoter region. In a family study, this *AHSP* variant, specifically the shorter homopolymer length (T_15_), was associated with reduced AHSP-mRNA and protein expression in an individual with thalassemia syndrome (genotype *β* ^0^ ^39–850 C-T^)/*β* ^N^, *α*Hb^*ααα*/*αα*^, and T_15_/T_18_), when compared with her mother (genotype *β* ^0 39–850 C-T^)/*β* ^N^, *α*Hb^*ααα*/*αα*^, and T_18_/T_18_) [9]. However, no significant differences in hematological parameters were found among *β*-thalassemia carriers from Southern China who were T_15_ homozygous (14 cases), T_18_ homozygous (223 cases) or T_15/T18 _heterozygous (128 cases) [39]. Conversely, Wang et al. showed that the introduction of a transgenic human-*AHSP* gene is able to neutralize excess *α*Hb in a murine model of *β*-thalassemia (*β* ^IVS-2-654^) and, as a consequence, ameliorate the phenotype of these animals [40]. Despite the partial improvement observed in the approach described, these data support the hypothesis that AHSP could be an adjuvant in gene therapy to treat *β*-thalassemia.

## 8. AHSP and *α*-Thalassemia-Like Syndromes

As stated previously, *AHSP* expression levels and protein function are important for the normal physiology of red blood cell production and could have a relevant impact on the phenotype of *β*-thalassemia. The level of *AHSP *mRNA from healthy individuals was not found to correlate with age or sex, but varied considerably, up to threefold in reticulocytes [9] and 10-fold in cultured erythroblasts [41]. This variability is probably associated with *AHSP* polymorphisms in regulatory regions since naturally occurring *AHSP* mutations that impair gene expression or protein function are rare [9, 10, 27].

The mutant AHSP^V56G^ described by Pissard et al. [42] is the first mutation in the *AHSP* gene that can be associated with *α*-thalassemia-like syndromes. The authors report the case of a child that presents with microcytic, hypochromic anemia, 3% of Hb Bart's, a normal globin gene pattern but a homozygous state for the  AHSP^V56G^ mutant. The parents and the first son were heterozygous carriers for this mutation and were clinically normal [42]. The clinical feature observed in the homozygous state may be explained by the altered biochemical and biophysical properties of the mutant protein and its association with an haplotype characterized by a decrease in AHSP expression [42, 43]. This mutation changes the Valine residue at position 56 to a Glycine. This residue is located at the corner between the two *α*-helices [39, 43]. Investigating the kinetic properties of the AHSP^V56G^, Brillet and coworkers demonstrated that despite its thermal instability (*T*_m_ of 42°C), the binding to *α*Hb and formation of the AHSP^V56G^-*α*Hb complex is not affected. The association rate constant is similar to that of AHSP^WT^ (*k*′ ≈ 20 *μ*M^−1^ s^−1^). In contrast, the AHSP^V56G^-*α*HB complex dissociates faster (dissociation rate of 0.5 s^−1^) than AHSP^WT^ (2 s-^1^), thus it seems that this reduced contact time does not allow the complex to complete the structural modifications required to stabilize *α*Hb chains [43].

 On the other hand, *α*Hb mutations may also result in impaired AHSP-*α*Hb interactions and could be responsible for thalassemia-like phenotypes without explanation in some patients. Indeed, this defective interaction has been shown for several *α*Hb variants, such as Hb Constant Spring and Pakse [44]; Hb Groene Hart [45, 46]; Hb Foggia [47], and several other *α*Hb variants [48, 49]. The structural abnormalities of these *α*Hb variants are located at the AHSP binding interface, therefore impairing their interaction. Failed interactions may lead to destabilization of native *α*Hb folding [48]. However, the mechanisms involved in this defective process are not well known, and further studies are required to better understand the biochemical and biological consequences of *α*Hb variants.

## 9. Summary and Perspectives

Molecular chaperones are essential for the maintenance of their target proteins. AHSP is only expressed in erythroid cells, specifically binds to *α*Hb, and does not bind to *β*Hb or HbA. As a molecular chaperone for *α*Hb, AHSP plays at least three important roles: (a), promotes proper folding of nascent *α*Hb, independently of the presence of iron, conferring resistance to proteolytic digestion; (b), inhibits auto-oxidation of holo-*α*Hb, converting the bound heme iron to an inert hemichrome structure (c) it promotes refolding of the denatured protein. As such, AHSP seems to be essential for normal erythropoiesis. Impaired up-regulation of AHSP leads to premature erythroid cell death, resulting in ineffective erythropoiesis. Reduced *AHSP* mRNA expression has been associated with clinical variability in some cases of *β*-thalassemia. It has been shown that *α*Hb variants can also impair AHSP-*α*Hb interactions, leading to pathological conditions that resemble *α*-thalassemia syndromes. As such, *AHSP* mutations may be considered when clinical severity is not explained by the *β*-thalassemia genotype, or in undiagnosed *α*-thalassemia-like syndromes.

Whilst available evidence strongly suggests that AHSP promotes the synthesis of HbA in red blood cells, future studies will help to better understand the molecular interactions of hemoglobin assembly and the relevance of these mechanisms in health and disease.
